# Evaluation of transrectal ultrasound-guided tru-cut biopsy as a complementary method for predicting pathological complete response in rectal cancer after neoadjuvant treatment: a phase II prospective and diagnostic trial

**DOI:** 10.1097/JS9.0000000000001152

**Published:** 2024-02-09

**Authors:** Yaoyi Huang, Yumo Xie, Puning Wang, Yao Chen, Si Qin, Fangqian Li, Yuanhui Wu, Mingzhe Huang, Zehui Hou, Yonghua Cai, Xiaosheng He, Hongcheng Lin, Bang Hu, Qiyuan Qin, Tenghui Ma, Shuyun Tan, Yi Liao, Jia Ke, Di Zhang, Sicong Lai, ZhiPeng Jiang, Huaiming Wang, Jun Xiang, Zerong Cai, Hui Wang, Xiaowen He, Zuli Yang, Donglin Ren, Xiaojian Wu, Yisong Hong, Meijin Huang, Yanxin Luo, Guangjian Liu, Jinxin Lin

**Affiliations:** a Department of General Surgery, The Sixth Affiliated Hospital, Sun Yat-sen University Guangzhou, China; b Medical Ultrasonics; c Radiology; d Guangdong Provincial Key Laboratory of Colorectal and Pelvic Floor Diseases, The Sixth Affiliated Hospital, Sun Yat-sen University; e Biomedical Innovation Center, The Sixth Affiliated Hospital, Sun Yat-sen University

**Keywords:** Neoadjuvant therapy, pathological complete response, rectal cancer, TRUS-TCB

## Abstract

**Importance:**

Patients with pathological complete response (pCR) of rectal cancer following neoadjuvant treatment had better oncological outcomes. However, reliable methods for accurately predicting pCR remain limited.

**Objective:**

To evaluate whether transrectal ultrasound-guided tru-cut biopsy (TRUS-TCB) adds diagnostic value to conventional modalities for predicting pathological complete response in patients with rectal cancer after neoadjuvant treatment.

**Design, setting, and participants:**

This study evaluated data of patients with rectal cancer who were treated with neoadjuvant treatment and reassessed using TRUS-TCB and conventional modalities before surgery. This study is registered with ClinicalTrials.gov.

**Main outcomes and measures:**

The primary outcome was accuracy, along with secondary outcomes including sensitivity, specificity, negative predictive value, and positive predictive value in predicting tumour residues. Final surgical pathology was used as reference standard.

**Results:**

Between June 2021 and June 2022, a total of 74 patients were enroled, with 63 patients ultimately evaluated. Among them, 17 patients (28%) exhibited a complete pathological response. TRUS-TCB demonstrated an accuracy of 0.71 (95% CI, 0.58–0.82) in predicting tumour residues. The combined use of TRUS-TCB and conventional modalities significantly improved diagnostic accuracy compared to conventional modalities alone (0.75 vs. 0.59, *P*=0.02). Furthermore, TRUS-TCB correctly reclassified 52% of patients erroneously classified as having a complete clinical response by conventional methods. The occurrence of only one mild adverse event was observed.

**Conclusions and relevance:**

TRUS-TCB proves to be a safe and accessible tool for reevaluation with minimal complications. The incorporation of TRUS-TCB alongside conventional methods leads to enhanced diagnostic performance.

## Introduction

Highlights

- Transrectal ultrasound-guided tru-cut biopsy proves to be a safe and accessible tool for pre with minimal complications.
- The incorporation of transrectal ultrasound-guided tru-cut biopsy alongside conventional methods significantly improved diagnostic accuracy compared to conventional modalities alone.

After neoadjuvant treatment for rectal cancer, ~15–20% of patients who receive such treatment can achieve a pathological complete response (pCR), characterized by the absence of tumour cells in surgical specimens. This outcome typically indicates improved oncology prospects^1–3^. In 2004, Habr-Gama *et al.* proposed that surgical resection might not significantly enhance overall and disease-free survival in these patients. This approach could instead increase the risk of surgical complications and necessitate a permanent stoma^4^. Consequently, they introduced the concept of “watch-and-wait” management for patients exhibiting clinical complete response (cCR), involving strict follow-up and observation without immediate surgery. A series of studies have demonstrated promising survival rates among patients who underwent nonoperative treatment, with 5-year overall survival and disease-free survival rates ranging from 85 to 93% and 82 to 94%, respectively. Furthermore, salvage surgery in case of relapse has shown success rates of 80–91%^2,5^.

However, there exists no standardized definition of clinical complete response that reliably predicts pCR. Common methods for primary re-assessment include digital rectal examination (DRE), fludeoxyglucose positron emission tomography, computed tomography, MRI, transrectal ultrasound (TRUS), and endoscopy. Among these techniques, MRI has exhibited superior performance and accuracy. Yet, MRI also displays a notable false-positive rate, leading to overestimation of tumour residues and unnecessary surgical interventions^6–8^. The determination of pCR post-neoadjuvant treatment for distal rectal cancer remains a challenge for clinicians.

In recent years, growing interest has emerged in MRI tumour regression grade (mrTRG) to enhance clinical response assessment. This approach relies on the characteristic MRI signal intensities of treatment-induced fibrosis (low intensity) and residual tumour (intermediate intensity). Its standardized protocol allows for a more precise and consistent evaluation of treatment-induced changes between clinicians and institutes^9^. Furthermore, in the aspect of pathology, Duldulao *et al.* ^10^ observed that post-neoadjuvant treatment, rectal tumour cells predominantly reside in the muscularis of the bowel wall, with fewer present in the mucosa. As a result, endoscopic forceps biopsies prove inadequate^7^, and full-thickness biopsies offer a more reliable sampling method to confirm malignancy across all stages of rectal cancer post-neoadjuvant treatment. Therefore, transrectal ultrasound-guided tru-cut biopsy (TRUS-TCB), capable of obtaining samples from the entire bowel wall layer, presents potential diagnostic utility for patients achieving pCR after neoadjuvant treatment. Despite its wide clinical use, there remains a scarcity of studies investigating the predictive value of TRUS-TCB in assessing pCR following preoperative therapy for rectal cancer^11–13^.

To this end, we conducted a phase II prospective study designed to assess tumour response through the integration of TRUS-TCB and conventional evaluations among rectal cancer patients after neoadjuvant therapy. By systematically analyzing the performance of TRUS-TCB combined with conventional examinations (mrTRG and colonoscopy) and conventional examinations, we aim to provide valuable insights that can facilitate the accurate prediction of pCR outcomes. Our study seeks to shed light on the potential of TRUS-TCB as a reliable method for anticipating pCR following neoadjuvant therapy for rectal cancer.

## Methods

### Study design, patients and outcomes

**Figure 1:**
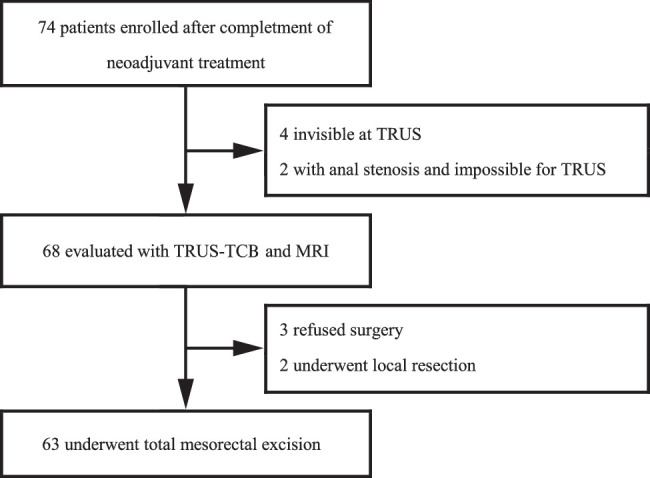
Study flowchart. TRUS, transrectal ultrasound.

Between June 2021 and June 2022, we conducted a phase II, open-label, single-centre, single-arm study. This study was conducted in accordance with the principles of the Declaration of Helsinki and Good Clinical Practice. Our work has been reported in the line with STARD^14^. Supplemental Digital Content 1, http://links.lww.com/JS9/B839. The key inclusion criteria were as follows: (i) locally advanced (T3-4N0 or TanyN1-2), resectable, histologically confirmed rectal adenocarcinoma; (ii) primary rectal cancer located within 10 cm from the anal verge; and (iii) complete full dose of neoadjuvant treatment. Written informed consent was obtained from all participants before enrolment. The central ethics committee of our hospital approved this experimental protocol. The flowchart of the study is shown in Fig. 1. The primary outcome of this study was the accuracy in predicting pCR. The secondary outcomes included sensitivity, specificity, negative predictive value (NPV) and positive predictive value (PPV).

### Procedure

#### Neoadjuvant treatment

Neoadjuvant treatments include chemotherapy alone (nCT) or concurrent with long-course external beam radiation therapy (nCRT), or immunotherapy. Radiotherapy consisted of 45–54 Gy delivered to the primary tumour and pelvic lymph nodes at risk. After completion of neoadjuvant treatment, patients were re-evaluated by TRUS-TCB, MRI and colonoscopy. For patients received chemoradiotherapy, the interval was at an interval of 6–8 weeks after completion of treatment; for those received chemotherapy or immunotherapy alone, it was 2–4 weeks.

#### Transrectal ultrasound-guided tru-cut biopsy

Before TRUS-TCB, the patients received routine cleaning enema using sodium phosphate enema. Four experienced sonographers performing TRUS-TCB were trained by standard protocol before trial. The patients were placed in the left lateral position. TRUS procedures were conducted using the BK Profocus2202 ultrasound diagnostic instrument (BK, Denmark) paired with an 8848 probe (frequency 4-13MHz), which allows simultaneous transrectal two-dimensional (2D) ultrasound and transrectal 360-degree annular ultrasound. This enables a thorough evaluation of the suspected area of abnormality and layers of involvement from the upper third of the rectum to the anal verge. Sonographers solely referred to TRUS results to determine the intramural mass lesions. Once these mass lesions were identified and characterized, a wideband microconvex intercavity array transrectal ultrasound probe, (GE LOGIQ E9, IC5-9-D) with 18 − G/25 cm core-cut biopsy needles was used for TRUS − TCB. At least three biopsy samples were obtained from suspicious sites in each patient. Biopsy specimens were immediately fixed in formalin, embedded in paraffin, dissected into sections, stained, and examined by experienced pathologists. After the TRUS-TCB, gauze was compressed into the anus to avoid bleeding and was removed the next day if there was no obvious bleeding. The patients were followed closely to avoid any biopsy-related complications prior to radical surgery.

#### MRI assessment

All enrolment were performed pelvis MR in our hospital. MR images were obtained using three devices: (1) 1.5T superconducting scanner magnetic resonance (Optima 360, GE Healthcare); (2) 3.0T superconducting scanner magnetic resonance (Discovery 750W, GE Healthcare); (3) 1.5T superconducting scanner magnetic resonance (uMR570, Shanghai United Imaging Healthcare). Sagittal mages with T2-weighted fast spin echo followed by oblique axial and oblique coronal T2-weighted images (perpendicular to and parallel to the long axis of the bowel wall where the rectal tumour was located, respectively), then followed by oblique axial diffusion weighted imaging, and contrast-enhanced T1-weighted images. The slice thickness was 3 mm, and the interslice gap was 0.5 mm for images. MRI-assessed T staging of tumour post treatment was based on interpretation of local extent of persistent tumour signal intensity relative to the layers of bowel wall on T2-weighted images.

#### MRI tumour regression grade

Anonymized MRI images were analyzed by two independent reviewers, who were blinded to clinical data and pathologic results. If the two reviewers drew different conclusions, the images were analyzed by a third reviewer to reach a consensus. Based on the re-assessment findings of MRI, patients were further classified into five grades using the mrTRG system proposed by the MERCURY study group^9^ (eFigure 1 in Supplement, Supplemental Digital Content 2, http://links.lww.com/JS9/B838): grade 1, the absence of tumour signal; grade 2, small amounts of residual tumour visible but with a predominant fibrotic low signal intensity; grade 3, mixed areas of low signal fibrosis and intermediate signal intensity present but without predominance of tumour; grade 4, predominantly tumour signal intensity remains with minimal fibrotic low signal intensity; grade 5, no fibrosis evident, tumour signal visible only.

#### Colonoscopy

A long flexible tube (colonoscope) is inserted into the intestinal lumen and reach caecum at the end. The catheter has a tiny camera at the tip that allows doctors to visualize the entire lumen and assess the rectal tumour. During the examination, a superficial biopsy would be performed when the tumour displays a good response without visible lesions or only shows flat scars, telangiectasia, or whitening of the mucosa.

#### Surgery

Patients who received chemoradiotherapy underwent radical rectal cancer resection at a mean interval of 6–8 weeks after the completion of treatment, and the interval between those who received chemotherapy or immunotherapy alone was 2–4 weeks. Operations followed the principles of total mesorectal excision (TME), including abdominal-perineal resection or low anterior resection with colorectal or coloanal anastomosis. Diverting loop ileostomies were performed in patients with coloanal anastomosis. All surgical resection specimens were sent for final pathology testing.

#### Histopathologic assessment

Histopathology of surgical resection specimens was the standard of reference. All surgically removed specimens were evaluated by experienced pathologists based on standard histologic analysis that included TNM classification according to the 2016 American Joint Committee on Cancer TNM system^15^ and pathological response after neoadjuvant treatment using the National Comprehensive Cancer Network tumour regression grade (TRG) scoring system. Pathological complete response was defined as no evidence of malignancy, which was ypT0.

#### Criteria for clinical complete response

For a single evaluative modality, the absence of tumour evidence—like no evidence of malignancy found in pathological examination and absence of any tumour signal on MRI, was considered as cCR. For the combination of conventional modalities, a mrTRG grade less than or equal to 2 and no malignancy found in superficial biopsy were considered as cCR. For combination of TRUS and conventional modalities, no malignancy found either in superficial TRUS biopsy or superficial biopsy and a mrTRG grade less than or equal to 2 were considered as cCR.

### Sample size and statistical analysis

We calculated that a sample size of 60 patients with 10% of dropout included would provide a power of 80% to detect an estimated improvement in the accuracy after adding TRUS-TCB in evaluation of tumour residues from 50 to 70% at a two-sided α of 0.05. The sensitivity, specificity, negative predictive value, positive predictive value, accuracy and 95% CIs were calculated. Continuous variables are presented as mean with standard deviation, and frequencies and percentages for categorical variables. The R package “DTComPair” (v1.2) were used to compare the diagnostic value of the examinations. Briefly, due to the number of patients with differing results is relatively small, we used the exact binomial test for comparing the differences in sensitivity and specificity, the generalized score statistic for the comparisons of predictive values, and McNemar’s test for the comparisons of accuracy, to ensure the robustness of analyses^16^. A value of *P* less than 0.05 was considered statistically significant. PASS 11 software (NCSS Statistical Software) was used to calculate the sample size. Statistical Package for the Social Sciences (SPSS) version 19. 0 (SPSS Inc.) and R v4.2.0 was used for analyses.

## Result

### Characteristics of the study population

The study comprised a total of 74 enroled patients, with 11 individuals excluded for various reasons: six due to not undergoing TRUS-TCB (two with intestinal stenosis and four with an indiscernible tumour under TRUS), three declining surgery, and two opting for local resection based on negative TRUS-TCB results and positive imaging response. Importantly, among the non-surgical patients, three remained alive and disease-free after a rigorous 8-month follow-up. The subsequent analysis centred on the remaining 63 patients. Demographically, the cohort consisted of thirteen females (21%) and fifty males (79%), ranging in age from 17 to 85 years (mean age 55.2 years). Median distance from the anal verge to the distal tumour margin was 5.1 cm (range 1.9–10.0 cm). Of the patients, twenty-five received neoadjuvant chemotherapy alone, while thirty-eight underwent concurrent neoadjuvant long-course radiation therapy; one individual received immunotherapy.

For the chemoradiation group, TRUS-TCB and surgery were performed at a median of 49 days and 54 days, respectively, after completion of chemoradiation treatment. The median interval between TRUS-TCB and surgery was 5 days. For the chemotherapy group, the median interval from completion of chemotherapy to surgery was 31 days, to biopsy was 27 days, and from biopsy to surgery was 4 days. One patient who received immunotherapy had the biopsy and surgery 14 days and 15 days after the completion of immunotherapy, respectively.

All 63 patients underwent surgery, adhering to total mesorectal excision principles. The surgical procedures included low anterior resection with temporary ileostomy in thirty-two patients (51%) and abdominoperineal resection with permanent sigmoid colostomy in eleven patients (17%). Notably, seventeen patients (29%) achieved a pCR: five after nCT, eleven after nCRT, and one following immunotherapy. The pCR rates were 29% and 22% within the nCRT and nCT groups, respectively.

These clinical characteristics are summarized in Table 1.

**Table 1:** Baseline demographic and clinical characteristics of the study population.

Variable	*N*
Sex, female/male	13/50
Distance from the anal verge, mean^a^, cm	5.1 (1.9–10)
Neoadjuvant treatment	
nCRT	39
nCT	23
Immunotherapy	1
Operations	
APR	11
LAR with stoma	32
LAR without stoma	20
Pathological T stage	
0	18
1	5
2	12
3	27
4	1
TRG	
0	18
1	7
2	33
3	5

APR, abdominoperineal resection; LAR, low anterior resection; nCRT, neoadjuvant chemoradiotherapy; nCT, neoadjuvant chemotherapy; TRG, tumour regression grade.

a Assessed by MRI.

### Diagnostic performance of evaluative modalities

Assessment of different evaluative modalities was performed by comparing their results with the final pathological status. Accuracy, sensitivity, specificity, NPV, and PPV in predicting tumour residues were analyzed and presented in Table 2.

**Table 2:** Summary of comparison between pathologic response and re-assessment modalities.

	TRUS-TCB	mrTRG
Sensitivity (95% CI)	0.61 (28/46, 0.45–0.75)	0.39 (18/46, 0.25–0.55)
Specificity (95% CI)	1.00 (17/17, 0.77–1.00)	0.94 (16/17, 0.70–1.00)
PPV (95% CI)	1.00 (28/28, 0.85–1.00)	0.94 (18/19, 0.71–1.00)
NPV (95% CI)	0.49 (17/35, 0.32–0.66)	0.36 (16/44, 0.23–0.52)
Accuracy (95% CI)	0.71 (45/63, 0.58–0.82)	0.54 (34/63, 0.41–0.66)

mrTRG, magnetic resonance imaging tumour regression grade; NPV, negative predictive value; PPV, positive predictive value; TRUS-TCB, transrectal ultrasound-guided tru-cut biopsy.

Given that clinicians would not rely on a single modality for the assessment of cCR in clinical practice, we combined the outcomes of TRUS-TCB and conventional evaluative methods, contrasting them with the results from conventional methods alone. Integration of TRUS-TCB significantly enhanced diagnostic performance (Table 3). The combined group exhibited an accuracy of 0.75 (95% CI 0.63–0.84), markedly surpassing that of the conventional group with 0.59 (95% CI 0.46–0.70, *P*=0.02). Sensitivity was notably elevated in the combined group compared to the conventional group (0.68 vs. 0.46, *P*=0.02). Furthermore, the NPV demonstrated improvement in the combined group over the conventional group (0.52 vs. 0.39, *P*=0.02). That is, forty-one patients were diagnosed as clinically complete or near-complete by conventional examination, and among these, 39% (16 patients) were confirmed as pCR. When TRUS-TCB was added, thirty patients were diagnosed as clinically complete or near-complete response, among which the accuracy of diagnosing pCR was thereby increased to 52%

**Table 3:** Diagnostic performance of each group.

	Combinational group	Conventional group	*P*
Sen (95% CI)	0.68 (31/46, 0.53–0.80)	0.46 (21/46, 0.31–0.60)	0.02
Spe (95% CI)	0.94 (16/17, 0.82–1.00)	0.94 (16/17, 0.82–1.00)	1.00
PPV (95% CI)	0.97 (31/32, 0.90–1.00)	0.95 (21/22, 0.86–1.00)	0.34
NPV (95% CI)	0.52 (16/31, 0.34–0.69)	0.39 (16/41, 0.24–0.53)	0.02
Acc (95% CI)	0.75 (43/63, 0.63–0.84)	0.59 (37/63, 0.46–0.70)	0.02

Acc, accuracy; NPV, negative predictive value; PPV, positive predictive value; Sen, sensitivity; Spe, specificity.

No significant difference in TRUS-TCB accuracy was found between patients with shorter and longer biopsy intervals after chemoradiation (*P*=0.07), and no significant difference was found for chemoradiation *(P*=0.54).

**Figure 2:**
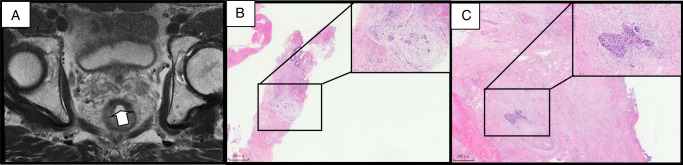
Rectal tumour response after neoadjuvant treatment. (A) Slides in the sagittal section of the MRI showed linear fibrotic low signal intensity. (B) Representative hematoxylin and eosin-stained slides of transrectal ultrasound-guided tru-cut biopsy showing tumour cells in all layers of the rectal wall. (C) Representative hematoxylin and eosin-stained slides of the final surgical specimen showing fragment tumour cells in the obvious fibrotic muscularis.

Thirty-one surgically confirmed non-pCR patients were detected by the combination group. Among these, 48% (15 patients) were detected by both TRUS-TCB and mrTRG, 42% (13 patients) by TRUS-TCB alone, and 10% (3 patients) solely by conventional evaluations. Namely, about 52% of patients (13/25) falsely diagnosed as cCR by prevailing clinical evaluative modalities, including mrTRG and superficial biopsy via colonoscopy, were subsequently revealed to possess tumour residues through TRUS-TCB. Moreover, even under the strictest criteria (without any signs in MRI), two patients previously diagnosed with cCR by conventional evaluations were confirmed to have residues through comprehensive whole-layer biopsy via TRUS-TCB (Fig. 2). These findings underscore the pronounced potential of TRUS-TCB to augment accuracy, sensitivity, and NPV in diagnosing tumour residues when integrated with conventional clinical evaluative methods, and its capability in detecting residual tumour after neoadjuvant therapy.

### TRUS-TCB demonstrates minimal complications

The majority of patients exhibited no complications following TRUS-TCB. A solitary case reported post-procedure bleeding, which was promptly controlled through intra-anal compression with gauze, infusion with a haemostatic agent, and transfusion. The cause of bleeding was unclear. The exact cause of bleeding remained indistinct. Thorough review by our sonographer confirmed the absence of any injured vessels within the needle tract zone suggesting inadvertent injury during the biopsy. Throughout the study, no additional complications were observed.

## Discussion

Our study findings underscore the substantial enhancement in diagnostic accuracy for detecting tumour residues in rectal cancer post-neoadjuvant therapy through the integration of TRUS-TCB with conventional evaluative techniques. Notably, the safety of TRUS-TCB was reaffirmed by the observation of a single adverse event. Remarkably, this study represents the largest prospective investigation to date, assessing the predictive capability of TRUS-TCB in determining pCR following neoadjuvant treatment.

Recent meta-analyses have revealed MRI’s specificity for predicting tumour residues to be 31%^6^—a result that aligns closely with our findings of 35%. This congruence highlights a notable portion of lesions detected by MRI that are, in fact, false-positive. Regrettably, this leads to the unwarranted undertaking of surgical procedures for patients with false-positive outcomes, entailing potential physical, emotional, and financial repercussions. Some studies have advocated for the utility of mrTRG in predicting pCR preoperatively^17^. However, results of meta-analysis demonstrated it only partial aligned with the final pathological status, yielding a pooled specificity of 32.3%^18^. A major constraint in accurately discerning residual tumours lies in the histopathological transformations of rectal lesions subsequent to neoadjuvant treatment—encompassing fibrosis, vascular proliferation, oedema, inflammation, and residual tumour lesions^7,19–21^.

TRUS-TCB holds a distinct advantage in harvesting the whole-layer biopsy of the rectal lesion, thus circumventing the potential pitfalls of tumour redistribution and concealing within adjacent normal mucosa^22^. Notably, the applicability of TRUS-TCB aligns seamlessly with lower rectal cancer cases, a cohort often preoccupied with sphincter preservation concerns, rendering it a fitting re-assessment tool. In our study, TRUS-TCB demonstrated a remarkable specificity of 1.00 (95% CI 0.77–1.00) and an accuracy of 0.71 (95% CI 0.58–0.82) when employed solely to assess tumour residues. This outperforms superficial biopsies under colonoscopy, as reported in prior studies with an accuracy of 0.45^7^. Furthermore, integration of TRUS-TCB with conventional modalities yielded significantly enhanced overall performance. Impressively, TRUS-TCB successfully differentiated ~50% (13 out of 25) of patients erroneously classified as cCR by conventional methods, showcasing its robust capability and vital role as a supplementary component to existing evaluative techniques. Given its marked safety profile and high accessibility, incorporating TRUS-TCB as a post-neoadjuvant evaluative modality holds immense promise.

Recent strides in employing neoadjuvant immunotherapy for patients with locally advanced rectal cancer characterized by microsatellite instability-high/deficient mismatch repair (MSI-H/dMMR) have yielded impressive outcomes^23–26^. However, conventional imaging results often diverge from actual pathological responses due to distinct immunotherapy response patterns compared to traditional chemoradiotherapy^23,25,26^. Thus, robust pathological evidence holds paramount importance for guiding clinical decisions in this context^27^. Beyond superficial biopsies, TRUS-TCB provides comprehensive whole-layer pathological insights. Combined with superficial biopsies, TRUS-TCB aids clinicians in “watch-and-wait” determinations for suspicious post-immunotherapy lesions, contributing to sphincter preservation^28^. A trial participant in our study receiving immunotherapy echoed previous findings—TRUS-TCB predicted pCR accurately, while MRI indicated residue. With neoadjuvant immunotherapy poised to reshape MSI-H/dMMR locally advanced rectal cancer treatment, TRUS-TCB stands as a pivotal tool for clinical decisions and potentially facilitated organ preservation.

This study does exhibit certain limitations. Initially, there is the potential for bias due to the inability to conduct analyses on all enroled patients, given the three individuals who declined TME surgery post-re-assessment. However, it is pertinent to note that these three patients displayed negative biopsy outcomes from TRUS-TCB and maintained disease-free status through ~6 months of rigorous follow-up. Consequently, the NPV of TRUS-TCB could possibly have been underestimated. Besides, we have to note that neoadjuvant treatment encompassed three treatment strategies which might have introduced some bias. However, subgroup analyses revealed that no significant difference of the diagnostic accuracy between patients received chemoradiotherapy and chemotherapy alone (0.77 vs. 0.70). Additionally, the study’s single-centre nature and the relatively confined patient population might have introduced bias, warranting consideration when interpreting the results.

In summary, our study underscores the safety and accessibility of TRUS-TCB as a valuable re-assessment tool, and combined with the results of current modalities can achieve a better prediction accuracy, thus, should be actively considered when imaging modalities cannot draw an affirmative conclusion. When combined with existing modalities, it exhibits improved predictive accuracy, making it an essential consideration when imaging results are inconclusive. This finding carries substantial promise, particularly as achieving pCR could translate to anal preservation and avoidance of permanent colostomy. Future investigations should be directed towards enhancing TRUS-TCB resolution, reducing device size, alleviating patient discomfort during examination, and exploring follow-up strategies for patients with negative biopsy outcomes.

## Ethics approval

Before conducting the study, we obtained approval from the review board of approval from the Sixth Affiliated Hospital of Sun Yat-sen University.

## Consent

Written informed consent was obtained from the patient for publication of this case report and accompanying images. A copy of the written consent is available for review by the Editor-in-Chief of this journal on request.

## Source of funding

We have made some changes to our source of funding: This work was supported by the National Natural Science Foundation of China (No. 82372715, YL; No. 81972245, YL; No. 82173067, YL; No. 82272965, HY; No. 82371966, GL), the Natural Science Foundation of Guangdong Province (No. 2022A1515012656, HY), the Fundamental Research Funds for the Central Universities, Sun Yat-sen University (2022007, MH), the Sixth Affiliated Hospital of Sun Yat-sen University Clinical Research-'1010' Program (1010CG(2022)-02, MH; 1010CG(2022)-03, YL; 1010PY(2022)-10, JL), the Medical Scientific Research Foundation of Guangdong Province (A2023094, JL), the Science and Technology Program of Guangzhou (202201011004, HY; 2023A04J1817, JL), the Scientific Research Project of the Sixth Affiliated Hospital of Sun Yat-Sen University (2022JBGS07), the Talent Project of the Sixth Affiliated Hospital of Sun Yat-sen University (No. P20150227202010251, YL), the Excellent Talent Training Project of the Sixth Affiliated Hospital of Sun Yat-sen University (No. R2021217202512965, YL), the Fundamental Research Funds for the Central Universities, Sun Yat-sen University (No. 23ykbj007, HY), the Program of Introducing Talents of Discipline to Universities (YL), and the National Key Clinical Discipline (2012).

## Author contribution

Conceptualization: G.L., J.L. Investigation, writing—original draft: Y.H., Y.X., Y.C., and P.W. Methodology: Y.H., Y.X., Y.C., P.W., S.Q., F.L., J.L., Y.L., G.L. Writing—review and editing: Y.H., Y.X., J.L., Y.L., G.L. Data acquisition: Y.H., Y.X., P.W., Y.C., S.Q., F.L., Y.C., X.H., H.L., B.H., Q.Q., T.M. S.T., Y.L., J.K., D.Z., S.L., Z.P.J., H.W., J.X., Z.C., H.W., X.H., Z.Y., D.R., X.W., Y.H., M.H., Y.L. Data analysis and interpretation: Y.H., Y.X., P.W., Y.C., S.Q., F.L. Study administration: Y.L., G.L., J.L. Final approval of publication: G.L., J.L.

## Conflicts of interest disclosure

The authors declare that they have no conflicts of interest.

## Research registration unique identifying number (UIN)

NCT04939103.

## Guarantor

Lin, and Liu had full access to all of the data in the study and take responsibility for the integrity of the data and the accuracy of the data analysis.

## Data availability statement

Data are available upon reasonable request.

## Provenance and peer review

Not commissioned, externally peer-reviewed.

## Supplementary Material
