# Supplementary material for: Evaluation of transrectal ultrasound-guided tru-cut biopsy as a complementary method for predicting pathological complete response in rectal cancer after neoadjuvant treatment: a phase II prospective and diagnostic trial
Source: Int J Surg. 2024 Feb 9;110(6):3230–6. doi: 10.1097/JS9.0000000000001152 (PMC11175734; doi:10.1097/JS9.0000000000001152)

**Legend**

**eFigure 1. mrTRG System Proposed by the MERCURY Study Group**

(A) mrTRG 1: the absence of any tumor signal; (B) mrTRG 2: small amounts of residual tumor visible but with a predominant fibrotic low signal intensity; (C) mrTRG 3: mixed areas of low signal fibrosis and intermediate signal intensity present but without predominance of tumor; (D) mrTRG 4: predominantly tumor signal intensity remains with minimal fibrotic low signal intensity; (E) mrTRG 5: no fibrosis evident, tumor signal visible only.


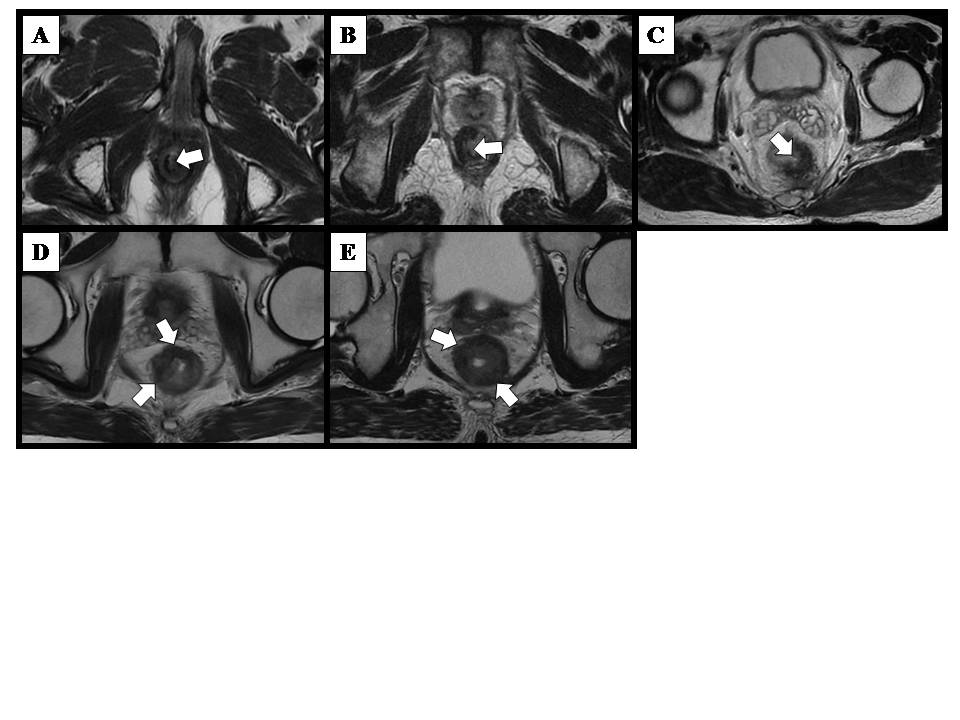

Supplement: Supplementary file 2 [file js9-110-3230-s002.docx]
